# Neutrophil Extracellular Traps in the Prognosis of Sepsis: A Current Update

**DOI:** 10.3390/medicina61071145

**Published:** 2025-06-25

**Authors:** Dimitrios Velissaris, Vasileios Karamouzos, Themistoklis Paraskevas, Eleni Konstantina Velissari, Charalampos Pierrakos, Christos Michailides

**Affiliations:** 1 Department of Internal Medicine, University Hospital of Patras, 26504 Rion-Patras, Greece; 2 Intensive Care Unit, University Hospital of Patras, 26504 Rion-Patras, Greece; vkaramouzos@hotmail.com; 3 Department of Nephrology, University Hospital of Patras, 26504 Rion-Patras, Greece; themispara@hotmail.com; 4 Department of Internal Medicine, Aigion General Hospital, 25100 Egio, Greece; elvelissari@yahoo.com; 5 Department of Intensive Care, Brugmann University Hospital, Université Libre de Bruxelles, 1050 Brussels, Belgium; charalampos.pierrakos@chu-brugmann.be

**Keywords:** sepsis syndrome, neutrophil extracellular traps, coagulopathy, NETosis, inflammation, citH3, MPO, cfDNA

## Abstract

Sepsis is a dysregulated host response to an infection characterized by the presence of coagulopathy and endothelial dysfunction. Neutrophil extracellular traps (NETs) are networks of extracellular fibers, primarily composed of DNA from neutrophils that bind invasive pathogens. These extracellular traps are involved in the activation and dysfunction of several pathways during the process of sepsis syndrome, including the immune response to injury, inflammation, and coagulation. Those formations consist of many molecules that have been studied as biomarkers for multiple sepsis pathophysiological pathways that reflect various complications. The best-studied segments of such formations, circulating free DNA, citrullinated histone 3 and myeloperoxidase, are considered to contribute to upscaling specificity. Plenty of NET end-products have been recently studied as indirect biomarkers for NET-related sepsis complications. Several studies have examined the relationship between NET end-products and established sepsis severity scores, such as Acute Physiology and Chronic Health Evaluation II (APACHE 2) and Multiple Organ Dysfunction Score (MODS). These studies also explore how these end-products contribute to the prognosis of acute respiratory distress syndrome (ARDS), mortality, and their efficacy in evaluating disseminating intravascular coagulation (DIC). This is a short review of the current literature regarding the evaluation of neutrophil extracellular trap levels in the prognosis of sepsis patients.

## 1. Introduction

NETs are implicated in the pathogenesis of many diseases characterized by endothelial dysfunction, such as sepsis and cardiovascular illnesses. In sepsis, the complex underlying pathophysiology, known as immunothrombosis, involves simultaneous activation of the inflammation and coagulation pathways, leading to growing interest in studying the role of NETs. NETs not only contribute to endothelial dysfunction but have also been found to exert procoagulant and prothrombotic activity through several mechanisms. NETs cascade end-products, including neutrophil elastase (NE), circulating free DNA (cfDNA, citrullinated histone 3 (citH3) and myeloperoxidase (MPO), not only play a crucial role in sepsis-related immune-regulated immunothrombosis and sepsis complications but could also serve as efficient biomarkers to predict unfavorable outcomes, such as mortality and ARDS development. Their correlation to commonly used prognostic scores could pave the way for further sepsis risk stratification. The utility of the use of such biomarkers involved in sepsis-associated immunothrombosis is still under investigation for their diagnostic and prognostic role.

## 2. Materials and Methods

### 2.1. Materials and Study Design

A systematic literature search of the PubMed database was conducted until May 2025 using the following search terms: “NETs” or “neutrophil extracellular traps” and “sepsis” or “sepsis prognosis” by two investigators and the derived literature was discussed and then interpreted.

### 2.2. Methods

Inclusion criteria included any type of study that referred to the prognostic significance of NETs for adult patients with sepsis. More precisely, we included studies based on the role of the NETs in the prognosis of patients suffering from confirmed or suspected sepsis syndrome. Exclusion criteria pertained to studies on pediatric patients, case reports, conference abstracts, theses, editorials, animal studies, duplicate studies and studies in a language other than English. From all studies, the following data were extracted: author, publication year and findings on the prognostic role of NETs in sepsis syndrome.

## 3. Results from Literature

A retrospective study by Su et al. evaluated the clinical data from 120 patients with sepsis admitted to a Chinese hospital for a two-year period. The studied patients were divided into three groups: the sepsis group, a 120-patient group with a common infection (bacterial group) and 120 healthy subjects (healthy group). Among various laboratory markers, NETs, coagulation and fibrinolysis indexes, prothrombin time (PT), fibrinogen (FIB), D-dimer level, international normalized ratio (INR) and disease severity scores (APACHE II and SOFA) in sepsis patients were compared to those in the bacterial and healthy groups. Correlations between these measures in all three groups were analyzed, and the predictive value of NETs for survival in patients with sepsis was assessed. Results showed that the levels of serum NETs, PT, FIB, D-dimer and INR value in sepsis patients were significantly elevated compared to the non-sepsis groups. The level of NETs was positively associated with APACHE II score, SOFA score, PT, FIB, D-dimer and INR. The authors concluded that the NETs and coagulation indexes have high predictive value for the prognosis of patients with sepsis [1].

A prospective study involving 82 sepsis patients in a critical care unit of a Chinese tertiary center revealed that, when NET formation was upregulated, there was a consequent sepsis-induced DIC incidence and mortality. In this study, clinical and hematological parameters and thrombotic or hemorrhagic events were recorded. Blood samples were obtained to assess components of NET formation that could serve as potential biomarkers, including neutrophil elastase 2 (ELA2) and citH3, as well as the endothelial-derived biomarker syndecan-1. NET interaction with autophagy regulation pathway was also examined, deducing that DIC was related to significantly higher levels of components associated with NET production and, vice versa, independently associated with DIC risk. Consequently, NET formation is closely related to DIC occurrence in the sepsis pathophysiological process [2]. Neutrophil elastase was found increased among sepsis patients compared to healthy volunteers and may reflect sepsis-induced acute lung injury (ALI) and ARDS [3].

A prospective pilot study from Germany investigated the predictive value of plasma neutrophil-derived cfDNA, a component of NETs, in the development of sepsis and mortality after patients had multiple traumas. Thirty-seven patients were finally assessed, and cfDNA plasma levels were directly measured. The time kinetics of cfDNA/NETs were compared to those of other biomarkers, such as C-reactive protein (CRP), interleukin 6 (IL-6), leukocyte counts and myeloperoxidase. The severity of the injury was calculated according to the Injury Severity Score; also, the sepsis scores MODS, SOFA and APACHE II were also calculated in the ICU. Elevated cfDNA/NET values (>800 ng/mL) were associated with worse events such as subsequent sepsis, multiple organ failure and death. IL-6 was significantly increased after admission, independently of a considered important second hit. CRP kinetics was not proved to have any correlation with sepsis development, in addition to cfDNA and NET kinetics. Circulating free DNA kinetics rather followed the kinetics of MODS and SOFA scores and leukocyte counts and partially that of myeloperoxidase. Circulating free DNA/NETs seems to append additional value for the calculation of injury severity and prediction of an inflammatory second hit in an intensive care unit (ICU) [4]. Per Jackson et al., citH3 and MPO increases reflect an increase in cfDNA in sepsis patients but not in trauma patients. Nevertheless, cfDNA was not found to correlate with MODS [5].

In a randomized control trial (RCT) by Qiao et al., plasma cfDNA and syndecan-1, which are used as NETosis markers, were found to be predictors of 28-day mortality at their baseline among patients with sepsis-induced ARDS. Thus, both markers were positively affected by high-dose intravenous vitamin C [6]. Another NETosis marker, aldehyde dehydrogenase 2 (ALDH2), was demonstrated to predict sepsis-related ARDS and mortality in mice. Moreover, MPO, a key NET formation marker, was stepwise increased among healthy volunteers, septic patients and patients with sepsis-induced ARDS [7]. Another study by Yang et al. revealed that septic patients with a hyperimmune phenotype undergo increased neutrophil activation and adhesion leading to NETosis, which is related to an increased need for mechanical ventilation and ICU length of stay (LOS) [8].

An observational study by Yokoyama et al. demonstrated that citH3 is positively correlated with organ failure in patients with infection. In the subgroup of patients with coagulopathy or cardiovascular failure, the increase was even sharper. A significant rise was also observed among patients with sepsis-induced DIC and non-survivors. Interestingly, citH3 was superior to CRP, WBC and cfDNA in predicting 28-day mortality in this cohort of patients with infection [9].

Another observational cohort study by Filippini et al., which included 1713 patients, examined the predictive role of histone 3.1 (H3.1) for sepsis and sepsis-related organ dysfunction, including acute kidney injury (AKI) and DIC. According to their findings H3.1 could discriminate septic from non-septic patients but the diagnostic accuracy was low (AUC = 0.59). Likewise, diagnostic accuracy for the prediction of sepsis-related organ failure was low to moderate for the prediction of ARDS, DIC and AKI (AUCs 0.59, 0.64 and 0.69, respectively). Interestingly, H3.1 was positively related to the severity of inflammation and the SOFA score [10]. Studies’ findings are summarized in Table 1.

An observational study by Morimont et al. compared NET markers among sepsis patients, COVID-19 patients and healthy controls. Nucleosome histone 3.1 (Nu.H3.1), citH3, NE, MPO and citrullinated histone H3R8 (Nu.Cit-H3R8) were examined in an ICU setting. All of these markers were statistically increased compared to the control group. Furthermore, Cit-H3/Nu.H3.1 and Nu.Cit-H3R8/Nu.H3.1 ratios and NE could discriminate critical viral infections from non-COVID-19 septic shock. MPO could correlate with APACHE and SOFA scores in septic patients in addition to COVID-19 patients [11]. Nu.H3.1 levels could also serve as early predictor of septic shock mortality [12].

Zhang et al. demonstrated that cfDNA and MPO are progressively more increased in infected patients and septic patients compared to healthy controls. Their increase offers an additional diagnostic value to CRP for sepsis diagnosis as CRP, cfDNA and MPO-DNA AUCs were 0.777 (95% CI: 0.680–0.873), 0.744 (95% CI: 0.650–0.838) and 0.719 (95% CI: 0.628–0.809), respectively, while the combination of them outperformed all biomarkers alone and all dual combinations as well with an AUC of 0.865 (95% CI: 0.795–0.919). Their levels were indicative for sepsis-related AKI, while MPO levels could also reflect myocardial and liver injury [13].

**Table 1:** Studies referring to NETs in prognosing sepsis.

First Author	Year of Publication	Type of Study	Major Findings
Su Y [1]	2023	Retrospective, single-center study, China, 120 patients	NET levels were related to APACHE II and SOFA scores and to biochemical indexes PT, FIB, D-dimer and INR. The NET and coagulation indexes have high predictive value for the prognosis of sepsis
Mao JY [2]	2021	Prospective, single-center study, China, 82 patients	Increased NET formation was significantly associated with sepsis-induced DIC incidence and mortality in sepsis patients
Margraf [4]	2008	Prospective pilot study with trauma patients in a German ICU, 37 patients	Circulating free DNA/NET kinetics followed kinetics of MODS and SOFA scores and leukocyte counts. Circulating free DNA/NETs seems to be a valuable additional marker for the prediction of inflammatory second hit in an ICU in trauma patients
Jackson [5]	2019	Retrospective study with ICU sepsis patients, Canada, 77 patients	citH3 and MPO reflect cfDNA increase in sepsis patients, but cfDNA is not correlated with MODS
Qiao [6]	2022	RCT with sepsis patients with ARDS, USA, 167 patients	cfDNA and syndecan-1 predicted 28-day mortality in patients with sepsis-induced ARDS
Yang [8]	2024	Observational study in critically ill ICU sepsis patients, USA, 45 septic patients, 7 healthy controls	NETosis is related to increased ICU LOS and need for mechanical ventilation
Yokoyama [9]	2019	Observational study in sepsis patients, Japan, 85 patients	citH3 is related to organ failure and can predict 28-day mortality
Filippini [10]	2025	Observational cohort study, Netherlands, UK, 1713 patients	H3.1 predicts sepsis and sepsis-related organ failure (ARDS, DIC, AKI) and is positively related to SOFA and inflammation markers
Morimont [11]	2022	Observational study, Belgium, 46 septic patients, 22 COVID-19 patients, 48 healthy controls	citH3, NE and MPO are important for the discrimination between viral and non-viral sepsis
Zhang [13]	2024	Observational study, China, 106 septic patients, 25 non-septic, 51 healthy controls	MPO and cfDNA levels could diagnose sepsis and sepsis-related organ dysfunction and displayed an additional diagnostic value to CRP for sepsis diagnosis

## 4. Discussion

Sepsis is now defined as a life-threatening organ dysfunction caused by the dysregulated response of the human body to infection [14,15]. This complex syndrome is characterized by the simultaneous activation of the inflammation and coagulation pathways, so-called immunothrombosis. The release of NETs has been identified as a part of the immune response in innate immunity. Neutrophil extracellular traps are composed of neutrophil-derived cfDNA and seem to be the most sensitive sepsis biomarker but rather specific compared to histones, such as citH3, and neutrophil cytoplasm-derived proteins such as MPO. This seems to reflect the earlier increase of cfDNA that triggers the citH3 formation and MPO activation. For this reason, the time of measurement may reflect their prognostic value [4,5,9]. NETs contribute to endothelial dysfunction and also exert procoagulant and prothrombotic activity through various mechanisms. NETs allow neutrophils to kill extracellular pathogens while minimizing damage to the host cells [4,5,7,16,17].

NETs serve as a scaffold and inducer of platelet adhesion, activation and aggregation, and this effect is mainly mediated by their specific components, histones H3 and H4. They also serve as scaffolds for red blood cells, promoting the generation of thrombi [18]. citH3 is released from NETs in blood and is a potential early biomarker for septic shock [19]. MPO and citH3 are NET formation markers. Ferritin and Mac-1 induce NET formation with the contribution of DNase-1, PAD4, NE and reactive oxygen species (ROS). Platelet activation, thrombi formation and inflammation are the results of this cascade. Sepsis-induced ALI may be caused by the interaction of NETs with the macrophage scavenger receptor in the same pathway [20,21,22].

Besides NET-induced platelet-dependent mechanisms of thrombin formation, NETs have been demonstrated to activate the intrinsic coagulation pathway and thus stimulate thrombin generation in plasma with low circulating platelets [23]. The interaction between NETs and membrane-derived microparticles released by activated neutrophils, an aftereffect of the sepsis cascade, enhances NET-mediated intrinsic coagulation pathway activation and subsequent thrombin formation, especially in microvessels. The induction of immunothrombosis is led by the activation of stimulator of interferon genes (STING) via Toll-like receptor 2 (TLR2) [24]. According to Shi et al., NETs in the immunosuppression sepsis phenotype upregulate Tregs, causing disturbances in cholesterol metabolism and presenting a potential drug target [25].

Sepsis-induced coagulopathy (SIC) is a life-threatening complication and its pathophysiology is explained by the systemic activation of coagulation cascade during the process of sepsis syndrome that affects those pathways. SIC is a frequent pathophysiological consequence of sepsis cascade that induces worse clinical outcomes, including increased length of hospital and ICU stay and significant associated mortality rates. Thus, the early detection of SIC is critical. Prolonged INR and reduced platelet count proceeding from sepsis spillover reflect SIC severity and happens due to increased activation of surface endothelial molecules and the aftershock of imbalance between anticoagulant and fibrinolytic pathways.

Biomarkers such as NETs involved in this pathophysiological procedure display both cytotoxic and procoagulant activities and have been investigated as prognostic tools [26]. Tissue factor is also an important molecule in this procedure as it seems to activate NET-related thrombin regulation [27]. Early identification of septic patients with high coagulopathy risks is of great importance as early anticoagulation and maybe DNAase I could serve as therapeutic tools in septic patients with NET-related coagulopathy [28]. Although acknowledged markers such as PT, APTT, D-dimers and platelets have been widely used to identify the development of DIC, those markers have low sensitivity in the early diagnosis of SIC and more accurate biomarkers such as NETs and NEUT-SFL could be helpful [29].

Some biomarkers that indicate sepsis-related endothelium injury and coagulation dysfunction have been used, such as thrombomodulin (sTM), thrombin–antithrombin complex (TAT), tissue plasminogen activator–inhibitor complex (t-PAIC) and α2-plasmin inhibitor–plasmin complex (PIC) [30]. NETosis can be suspected by the detection of NET components including citH3 and cfDNA in fluid samples. However, the gold standard marker for NETosis, or the method of NET detection, has not been established yet [31].

MODS is the most severe complication of sepsis progression and is highly correlated with worse prognosis. Excessive neutrophil extracellular traps are critical players in the development of organ failure during sepsis. Sepsis cardiomyopathy, encephalopathy and AKI are mediated by NET-associated oxidative stress. citH3, MPO and NE are potential biomarkers for sepsis AKI [32]. The molecules that are involved in this procedure, including estradiol, lactate, JQ1 and HMGB1, and the Wnt3/β-catenin/TCF4 signaling pathway are possible therapeutic targets [33,34,35,36]. Therefore, interventions targeting NETs’ release would likely effectively prevent NET-based organ injury associated with this disease.

Investigating several NETosis models and pathways, agents that could serve as therapeutic targets have emerged. Astragaloside seems to block NET release through blockage of the NF-κB pathway [37]. Mac-1 inhibition seems to stop NET formation though a crucial step in this immunological procedure, the prevention of histone 3 citrullination [38]. Further, if citH3 is simultaneously inhibited along with S100A8/A9, neutrophil activation is also downregulated [39]. Although the ideal biomarker with 100% sensitivity and specificity for diagnosis, treatment, and prognosis for sepsis has not yet been identified, several molecules have been studied in this direction. Based on the critical balance of endothelial injury and sepsis coagulopathy, the evaluation of NETs could serve as a possible tool for sepsis prognosis. According to our literature review, not many studies have been published, and few clinical data are available, despite the promising biological and clinical data regarding NETs and their components.

In comparison to other biomarkers, cfDNA and MPO provide an additional diagnostic value to CRP for sepsis diagnosis [13]. CRP is a well-established sepsis biomarker that can predict sepsis mortality and ICU LOS, especially with serial measurements [40,41]. Procalcitonin has also an established position as an antibiotic treatment monitoring biomarker, which can reduce antibiotic exposure and improve outcomes in infected patients [42,43]. Presepsin (PSEP) has a good diagnostic value for sepsis (AUC = 0.805) in the ED [44]. Consequently, PSEP outperforms NET-related markers (10, 13). Pancreatic stone protein is demonstrated to have an AUC = 0.694 for sepsis prediction in patients with intra-abdominal infection [45] and has an excellent diagnostic value for sepsis patients in combination with CRP that also outperforms NET-related markers and their combination with CRP [10,13,46].

## 5. Conclusions

Neutrophil extracellular traps provide a novel link between thrombosis and inflammation; for that reason, they have gained significant attention in the diagnosis, treatment and prognosis of sepsis syndrome. There is a need for determining the standard reference value range and to assess NETs’ possible role, in combination with other sepsis biomarkers and scores, for early diagnosis, better assessment of disease progression, identification of new therapeutic targets and improvement of prognosis. The upcoming increased use of NETs and their components as biomarkers of the sepsis process could give prominence to specific pathophysiologic procedures and serve as a tool for clinicians to better understand the phenotype of each individual patient. MPO, NE, cfDNA, citH3 and other NET-related biomarkers should be investigated in RCTs for their diagnostic and prognostic accuracy in sepsis and sepsis-related thrombotic events and other complications to define their cut-offs and diagnostic and prognostic value. Future studies, hopefully yielding positive results, are necessary to achieve this goal.

## 6. Future Directions

Unraveling the literature regarding NETs, there are points that need to be raised for further future clinical research. Since there is no ideal biomarker neither for sepsis nor for one of the most recently established and studied pathways, that of immunothrombosis, the position of NETs’ components is crucial for clinicians to identify this specific patient group. cfDNA, MPO and citH3 are some of the most studied and conveniently countable particles of this procedure. For this reason, there is the need to set their specific cut-offs, distinguish their role and estimate their diagnostic and prognostic accuracy with large-scale studies, which should include septic patients with immune-related thrombotic events, such as deep vein thrombosis and ischemic stroke. This would help to integrate them stepwise in risk stratification scoring along with other imaging and laboratory findings.
